# Surface-enhanced hyper-Raman and Raman hyperspectral mapping†
†Electronic supplementary information (ESI) available: SEM images, hierarchical cluster analysis of SEHRS data. See DOI: 10.1039/c6cp01625a



**DOI:** 10.1039/c6cp01625a

**Published:** 2016-05-04

**Authors:** Marina Gühlke, Zsuzsanna Heiner, Janina Kneipp

**Affiliations:** a Humboldt-Universität zu Berlin , Department of Chemistry , Brook-Taylor-Str. 2 , 12489 Berlin , Germany . Email: janina.kneipp@chemie.hu-berlin.de; b School of Analytical Sciences Adlershof SALSA , Humboldt-Universität zu Berlin , Albert-Einstein-Str. 5-9 , 12489 Berlin , Germany

## Abstract

We investigate distributions of crystal violet and malachite green on plasmonic surfaces by principal component analysis (PCA) imaging of surface-enhanced hyper-Raman scattering (SEHRS) data. As a two-photon excited Raman scattering process, SEHRS provides chemical structure information based on molecular vibrations, but follows different selection rules than the normal, one-photon excited surface-enhanced Raman scattering (SERS). Therefore, simultaneous hyperspectral mapping using SEHRS excited at 1064 nm and SERS excited at 532 nm improves spatially resolved multivariate discrimination based on complementary vibrational information. The possibility to map distributions of the structurally similar dyes crystal violet and malachite green demonstrates the potential of this approach for multiplex imaging of complex systems.

## Abstract 2

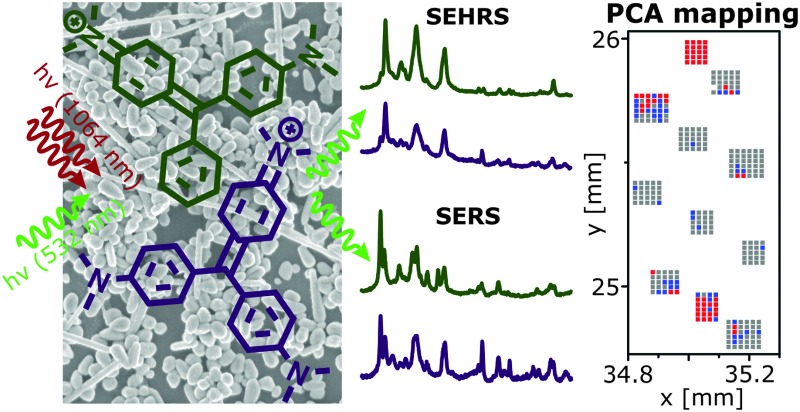
We investigate distributions of crystal violet and malachite green on plasmonic surfaces by principal component analysis (PCA) imaging of surface-enhanced hyper-Raman scattering (SEHRS) data.

## Introduction

SEHRS, the two-photon analogue of SERS can provide vibrational information different from that obtained in SERS due to different selection rules for one- and two-photon excited Raman transitions.^1^ As a non-linear Raman scattering process, SEHRS particularly benefits from the large signal enhancement that occurs in the local electromagnetic field at metal nanostructures.^2–5^ This enables measurements of SEHRS spectra at good signal to noise ratio in short collection times, despite extremely small cross sections for normal hyper-Raman scattering. In two-photon excited SEHRS, a high confinement of the probed volume also adds advantages in imaging, as millimeter-scaled areas can be mapped using focal volumes of ∼40 μm^3^, one order of magnitude smaller than in the one-photon excited case under the same excitation conditions.^6^ Among other advantages of two-photon excitation, such as the confined focal volume, reduced material damage, and great penetration depth, the detection upon excitation in the near-infrared is in the visible spectral range.

In SERS, the usage of multivariate tools has been shown to be powerful in the analysis of distributions of different analytes.^7–9^ Here, we present an approach for microscopic hyperspectral mapping of a surface covered by two structurally almost identical dyes based on PCA applied to SEHRS data. In a combination of SEHRS and SERS, the different selection rules for one- and two-photon excited Raman scattering provide the opportunity to gather complementary structural information of a sample.

## Materials and methods

**Fig. 1:**
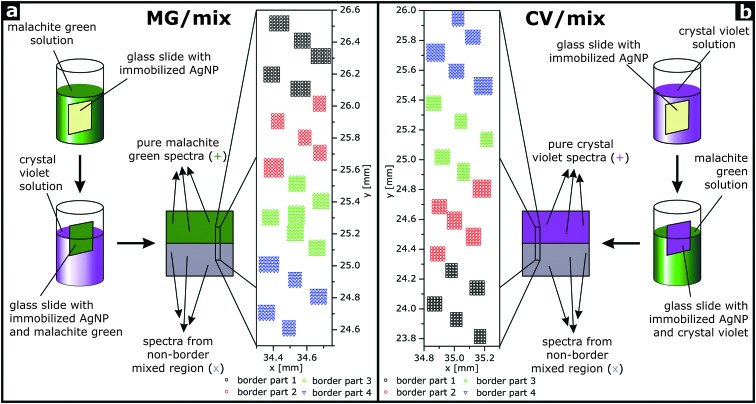
(a and b) Preparation procedures for the two types of samples containing either (a) a region with malachite green and a region with malachite green and crystal violet (MG/mix) or containing (b) a region with crystal violet and a region with crystal violet and malachite green (CV/mix). The arrows pointing from the sample schematically represent the places on the samples where SEHRS and SERS spectra were acquired. The millimeter-scaled border regions between the respective pure dye and the respective mixed region are magnified in a and b. They were divided into separate subregions (denoted ‘border part 1’ *etc.*) for later analysis. The step width in the smaller mapping grids is 20 μm. The schematic illustrates the samples discussed in Fig. 3–5.

Samples were fabricated by immobilizing citrate stabilized silver nanoparticles^10^ on glass slides using 3-aminopropyltriethoxysilane as a linker^11,12^ and immersing these slides in a macroscopic procedure in solutions of crystal violet (CV) and malachite green (MG). First, a slide was completely immersed in a 10^–6^ M solution of either MG or CV, respectively, for 30 minutes and, after drying, half of the slide was immersed in a 10^–6^ M solution of the respective second analyte for 30 minutes. This yielded samples termed MG/mix (Fig. 1a) and CV/mix (Fig. 1b), respectively. Upon dipping of the slides, the surface of the analyte solution is distorted, leading to a microscopic heterogeneity at the border between the two regions. These border regions, as well as the two distinct non-border regions (CV and mix or MG and mix, respectively) were investigated by SEHRS hyperspectral mapping. Several small areas with 20 μm distances between measurement points were put together to examine a range of approximately 2 mm across the border (Fig. 1a and b). To construct hyperspectral images, a PCA of the spectra from these regions, together with the spectra from the respective non-border regions in the same sample, was performed. To divide the spectra from the border region into three groups, two threshold values for the scores of the first principal component were defined. The threshold values were based on the scores of the non-border spectra in the following way: for the MG/mix sample, where score values from the two non-border regions overlap, border spectra with scores below the minimum score value of the non-border mixed spectra were defined as pure MG spectra, and border spectra with scores above the maximum score value of the non-border MG spectra were defined as mixed region spectra (see dashed lines in Fig. 3a and 4a). For the CV/mix sample, where the score values for the two non-border regions do not overlap, border spectra with scores below the maximum score value of the non-border mixed spectra were defined as mixed region spectra, and border spectra with scores above the minimum score value of the non-border CV spectra were defined as pure CV spectra (see dashed lines in Fig. 3c and 4c). For both samples, border spectra with scores between the two threshold values were put in a third group that cannot be assigned to either of the two regions.

**Fig. 2:**
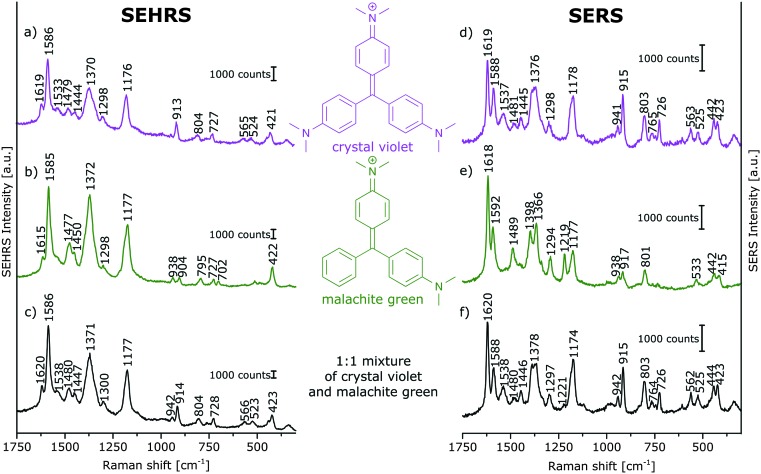
(a–c) SEHRS and (d–f) SERS spectra of aqueous solutions of (a and d) crystal violet, (b and e) malachite green, and (c and f) of a 1 : 1 mixture of crystal violet and malachite green on immobilized silver nanoparticles. Analyte concentrations: in (a and b) 10^–5^ M, in (c) 5 × 10^–6^ M for the single analytes, in (d and e) 10^–6^ M, in (f) 5 × 10^–7^ M for the single analytes. Excitation: 1064 nm, peak intensity: 10^10^ W cm^–2^, 30 s (a–c); 532 nm, peak intensity: 3 × 10^9^ W cm^–2^, 100 ms (d and e); 532 nm, peak intensity: 2 × 10^8^ W cm^–2^, 500 ms (f).

**Fig. 3:**
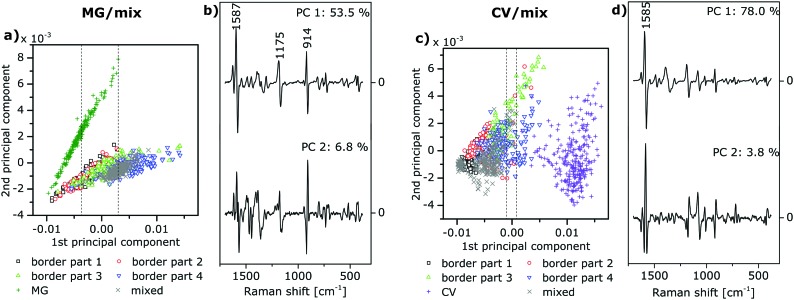
(a and c) Scores and (b and d) loadings of the first two principal components (PC) resulting from the principal component analysis (PCA) of the vector-normalized first derivatives of SEHRS spectra over the range 380–1700 cm^–1^ of (a and b) the MG/mix sample and (c and d) the CV/mix sample. Symbols and colors in a and c correspond to the subregions in Fig. 1a and b, respectively. Spectra from the two non-border regions on each sample (MG and mixed, respectively, in a, and CV and mixed, respectively, in c) were also included in the PCA and served as references. The dashed lines in a and c demarcate the ranges of score values of the first PC, which were used for the differentiation between different types of spectra in Fig. 5. Variance in percent that is explained by each PC is indicated in the loadings plots in b and d.

**Fig. 4:**
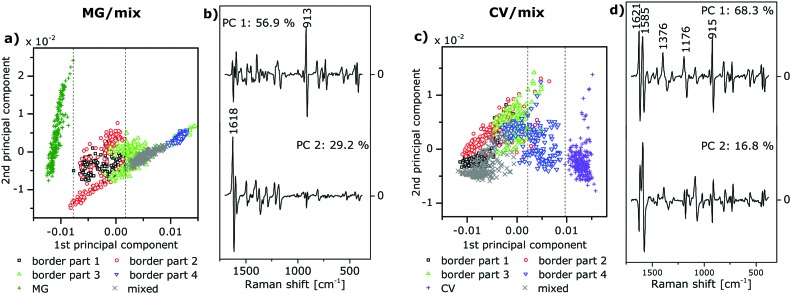
Results of the principal component analysis of the SERS spectra of (a and b) the MG/mix sample shown schematically in Fig. 1a, and (c and d) the CV/mix sample shown schematically in Fig. 1b. SERS spectra were measured on the same samples and in approximately the same positions on the samples as the SEHRS spectra, which are analyzed in Fig. 3.

The microscopic setup has been described previously.^6^ SEHRS spectra were excited at 1064 nm with a mode-locked laser (High Q Laser GmbH) with a pulse length of 7 ps and a repetition rate of 76 MHz with peak excitation intensities from 3 × 10^9^ to 1 × 10^10^ W cm^–2^ (average power at the sample ranging from 5 to 22 mW). SERS spectra were excited at 532 nm with the second harmonic of the same laser with peak excitation intensities from 2 × 10^8^ to 3 × 10^9^ W cm^–2^ (average power at the sample ranging from 0.2 to 4 mW). The scattering was collected with a 60× water immersion objective (NA = 1.2). Carrying out the experiment with a water immersion objective is necessary to achieve probing in small focal volumes and in addition provides an easy way for the dissipation of potential heat generated by the excitation radiation. CV and MG have electronic absorptions around 588 nm and 617 nm, respectively (spectra not shown), close to the second harmonic of the NIR laser, leading to additional resonance enhancement.

## Results and discussion

To investigate the potential of SEHRS in multivariate mapping of large areas in this initial study here, a well-known, well-defined type of plasmonic surface, immobilized silver nanoparticles that yield homogeneous SERS enhancement on the microscopic scale,^12^ were used for imaging experiments. Similarly, excitation in resonance was chosen to make sure that the signal to noise ratio was high enough at any point of the samples in spite of the inherently low scattering cross sections of SEHRS. Here, we prepared samples with distinguishable areas for SEHRS hyperspectral mapping by immersing slides with immobilized silver nanoparticles subsequently in solutions of CV and MG (Fig. 1). SEM images of the immobilized silver nanoparticles without dye molecules (Fig. S1a, ESI†) and after immersion in dye solutions (Fig. S1b, ESI†) show that the particle density is not changed in the presence of the dyes.

Prior to the imaging experiments, droplets of aqueous solutions of the pure dyes and of a mixture of the dyes were placed on the immobilized silver nanoparticles, and their SEHRS and SERS spectra were obtained (Fig. 2). CV and MG differ in one dimethylamino group and their SEHRS spectra (Fig. 2a and b) are almost identical except for some bands, in accord with a previous report.^13^ In particular, the in-plane phenyl deformation mode^14^ is found at 913 cm^–1^ for CV and at 904 cm^–1^ for MG with different intensity compared to the dimethylamino stretching band^14^ at 938 cm^–1^. By contrast, the shift of this band is not significant in the SERS spectra (Fig. 2d and e). SEHRS and SERS spectra of a mixed solution with equal concentrations of both dyes (Fig. 2c and f) are more similar to the spectra of pure CV than to those of MG, due to either a higher Raman scattering cross section or preference of CV in the interaction with the silver nanostructures. To utilize as many as possible of the several subtle differences between both the SEHRS and the SERS spectra of CV and MG, we apply multivariate methods to map the distribution of the dyes on surfaces prepared according to the schematics shown in Fig. 1.

A first similarity assessment by unsupervised hierarchical cluster analysis with the SEHRS spectra from all different samples (Fig. S2, ESI†), yields two main groups of spectra. They correspond to the spectra of CV and MG, respectively, and to mixture spectra that are similar to either of the two dyes.

For mapping, one-photon and two-photon excited spectra were analyzed with PCA. PCA and spectra pre-processing (calculation of vector-normalized first derivatives) were performed using MATLAB (The Mathworks, Inc.). The scores plot obtained as a result of a PCA indicates the formation of groups of spectra that are similar with respect to a specific principal component. The loadings provide information about those bands in the spectra that are responsible for the separation of the spectra with respect to the corresponding principal component. The scores of the PCA of the SEHRS spectra of an MG/mix sample yield clear separation of the MG spectra (Fig. 3a, green ‘+’) from the mixed spectra (Fig. 3a, grey ‘×’) mostly due to the variation represented by the first principal component (PC) with a small contribution of the second PC. The loadings (Fig. 3b) show a large variance in the spectra around 914 cm^–1^, where the SEHRS spectra of CV and MG display bands at different frequencies (see also Fig. 2a and b). The first PC also indicates differences around 1587 cm^–1^ and 1175 cm^–1^, where all of the SEHRS spectra show intense phenyl bands (Fig. 2a–c). For the CV/mix sample (Fig. 1b), separation of CV spectra (Fig. 3c, purple ‘+’) and spectra from the mixed region (Fig. 3c, grey ‘×’) is done by the first PC alone, and mainly caused by differences in the phenyl band at 1585 cm^–1^ (Fig. 3d, upper trace). Although the additional dimethylamino group in CV is the only difference between the two molecules, bands assigned directly to this group have less impact on the distinction of different proportions of the two dyes than the phenyl modes have. We ascribe this to a strong influence of the dimethylamino groups on the mesomeric system of the triarylmethane backbone, on the molecular symmetry, and the resulting changes in the interaction with the silver surface.

PCA of the SERS spectra, which were obtained from nearly the same positions as the SEHRS spectra, enables separation of the different regions of the samples in a similar way (Fig. 4).

**Fig. 5:**
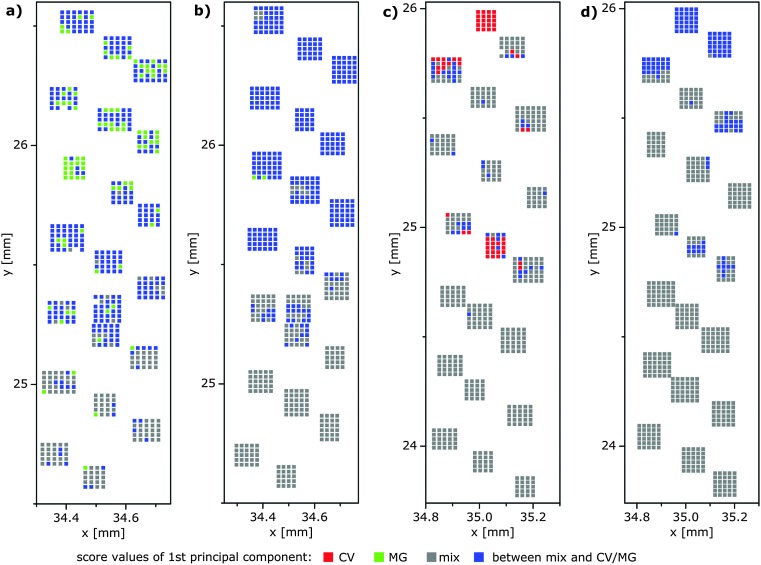
(a) False-color SEHRS and (b) SERS maps of the border region of the MG/mix sample shown schematically in Fig. 1a. (c) False-color SEHRS and (d) SERS maps of the border region of the CV/mix sample shown schematically in Fig. 1b. The maps are based on the scores of the respective first principle component of the PCA of (a and c) the SEHRS spectra (compare Fig. 3a and c, respectively) and (b and d) of the SERS spectra (see also Fig. 4a and c). The *x* and *y* axes of the maps display the absolute coordinates of the *x*,*y*-stage that was used for the two respective mapping experiments in order to demonstrate the overlap of the microscopic regions that were probed by SEHRS and SERS, respectively (in a and b and in c and d, respectively).

In the scores plots of the SEHRS and the SERS data (Fig. 3a and c and Fig. 4a and c), the spectra from the MG/mix and CV/mix border regions appear very close to the spectra from the non-border mixed regions (grey ‘×’ in all scores plots). Nevertheless, a gradual change of the score values across the border is observed for the first PC (Fig. 3a and c and Fig. 4a and c). This PC was used to reconstruct hyperspectral maps using both SEHRS and SERS data (Fig. 5). The color scale of the maps in Fig. 5 was set according to the score values of the spectra, arbitrarily separating the axis of the first PC into three ranges for every scores plot. Threshold score values for the separation are presented by the maximum and minimum score values of the spectra of the single analyte regions and the non-border mixed regions as described in detail in the Materials and Methods section (see dashed lines in Fig. 3a and c and in Fig. 4a and c). The two outer ranges cover those spectra that can be unequivocally assigned to a single-analyte region or to a mixed (non-border) region, respectively. In the third range in the middle, SEHRS spectra from the border, mixed, and single-analyte regions overlap (Fig. 3a and c). In the SERS data (Fig. 4a and c), this intermediate range contains only spectra from the border region. In the upper part of the border region of the MG/mix sample, which points towards the pure MG region (compare Fig. 1a), several SEHRS spectra are comparable to pure MG spectra (Fig. 5a, green pixels), while the rest of the SEHRS spectra and most of the SERS spectra in this part have score values within the middle range (Fig. 5a and b, blue pixels, Fig. 3a, and Fig. 4a). In the lower part of the map, more spectra are assigned to the mixed region (Fig. 5a and b, grey pixels). For the CV/mix sample, most spectra from the border are almost identical to the spectra in the mixed region (Fig. 5c and d, grey pixels). Some of the spectra in the upper part of the map, towards pure CV (compare Fig. 1b), are more similar to CV region spectra in SEHRS (Fig. 5c, red pixels) and have score values between distinct CV or mixed region spectra in SERS (Fig. 5d, blue pixels, Fig. 4c). In summary, the hyperspectral maps in Fig. 5 show a gradient from the single-analyte region to the mixed region. The differences within the border regions are very small.

It should be noted that the arbitrary separation of the score values based on the values for non-border spectra defined by the experiment and the principal component(s) chosen for imaging are specific for this particular imaging problem. For the application of this hyperspectral imaging approach to other sample systems, the parameters for the construction of the color scale have to be determined based on appropriate reference spectra. The fact that classification of pixels in the SEHRS maps can differ from the SERS maps (Fig. 5a and b) holds great promise to exploit the extensive chemical information of complementary SEHRS and SERS imaging. In particular, the possibility to characterize chemical interactions in nanoenvironments with SEHRS^6,15–18^ gains applicability now, *e.g.* for complementing existing SERS data about molecular interaction of proteins or drugs.^19,20^ In the future, this will be extended to the 2D mapping of such interactions. The high sensitivity of SEHRS converts HRS microscopy from the localization of a single strong resonant signal^21^ to the spatially resolved hyperspectral analysis in microstructured samples.

## Conclusions

In conclusion, we have shown simultaneous microscopic SEHRS and SERS imaging and multivariate discrimination of dyes on immobilized silver nanoparticles. PCA of the spectra revealed the spatial distribution of structurally very similar analytes and their mixtures on surfaces. In the example chosen here, the loadings obtained in the PCA of the SEHRS data indicate a high sensitivity towards small differences in the phenyl modes of the two very similar dyes. This suggests that discrimination between SEHRS spectra of more complex mixtures of molecules, and under non-resonant conditions, *e.g.*, in cells,^22^ should also be possible. The results provide evidence that SEHRS offers additional possibilities for multivariate discrimination and mapping based on complementary vibrational information. In the future, this approach will be used for multiplex imaging in complex systems.

## References

[cit1] Denisov V. N., Mavrin B. N., Podobedov V. B. (1987). Phys. Rep..

[cit2] Baranov A. V., Bobovich Y. S. (1982). JETP Lett..

[cit3] Golab J. T., Sprague J. R., Carron K. T., Schatz G. C., van Duyne R. P. (1988). J. Chem. Phys..

[cit4] Kneipp J., Kneipp H., Kneipp K. (2008). Chem. Soc. Rev..

[cit5] Itoh T., Ozaki Y., Yoshikawa H., Ihama T., Masuhara H. (2006). Appl. Phys. Lett..

[cit6] Gühlke M., Heiner Z., Kneipp J. (2015). Phys. Chem. Chem. Phys..

[cit7] Mamian-Lopez M. B., Poppi R. J. (2015). Microchem. J..

[cit8] Matschulat A., Drescher D., Kneipp J. (2010). ACS Nano.

[cit9] McAughtrie S., Lau K., Faulds K., Graham D. (2013). Chem. Sci..

[cit10] Lee P. C., Meisel D. (1982). J. Phys. Chem..

[cit11] Chumanov G., Sokolov K., Gregory B. W., Cotton T. M. (1995). J. Phys. Chem..

[cit12] Joseph V., Gensler M., Seifert S., Gernert U., Rabe J. P., Kneipp J. (2012). J. Phys. Chem. C.

[cit13] Schneider S., Brehm G., Freunscht P. (1995). Phys. Status Solidi B.

[cit14] Lueck H. B., Daniel D. C., McHale J. L. (1993). J. Raman Spectrosc..

[cit15] Kneipp J., Kneipp H., Wittig B., Kneipp K. (2007). Nano Lett..

[cit16] Kneipp H., Kneipp K. (2005). J. Raman Spectrosc..

[cit17] Nie S. M., Lipscomb L. A., Feng S., Yu N. T. (1990). Chem. Phys. Lett..

[cit18] Leng W. N., Kelley A. M. (2006). J. Am. Chem. Soc..

[cit19] Howes B. D., Scatragli S., Marzocchi M. P., Smulevich G. (2006). J. Raman Spectrosc..

[cit20] Lopez-Tobar E., Hernandez B., Gomez J., Chenal A., Garcia-Ramos J. V., Ghomi M., Sanchez-Cortes S. (2015). J. Phys. Chem. C.

[cit21] Shimada R., Kano H., Hamaguchi H. O. (2006). Opt. Lett..

[cit22] Kneipp J., Kneipp H., Kneipp K. (2006). Proc. Natl. Acad. Sci. U. S. A..

