# Supplementary material for: Surface-enhanced hyper-Raman and Raman hyperspectral mapping
Source: Phys Chem Chem Phys. 2016 May 4;18(21):14228–33. doi: 10.1039/c6cp01625a (PMC5043135; doi:10.1039/c6cp01625a)
Supplement: Supplementary file 1 [file CP-018-C6CP01625A-s001.pdf]

Electronic Supplementary Material (ESI) for Physical Chemistry Chemical Physics.
This journal is © the Owner Societies 2016

Surface-enhanced hyper-Raman and Raman hyperspectral mapping

Marina Gühlke[a], Zsuzsanna Heiner[a,b], Janina Kneipp*[a,b]

[a]Humboldt-Universität zu Berlin, Department of Chemistry, Brook-Taylor-Str. 2, 12489 Berlin, Germany, e-mail: janina.kneipp@chemie.hu-berlin.de

[b]School of Analytical Sciences Adlershof SALSA, Humboldt-Universität zu Berlin, Albert-Einstein-Str. 5-9, 12489 Berlin, Germany

Electronic Supplementary Information

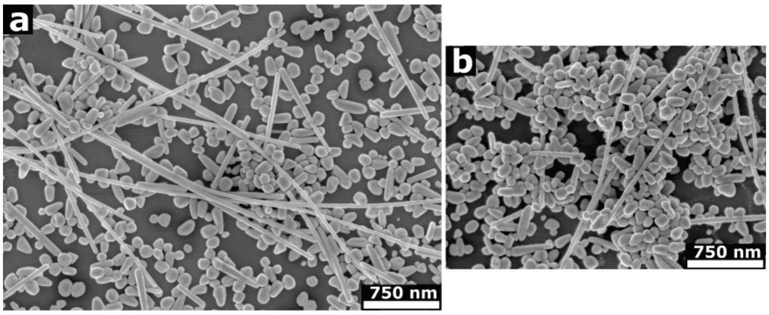

Fig. S1 SEM images of **(a)** immobilized silver nanoparticles without dye molecules and **(b)** of immobilized silver nanoparticles after sequential immersion in 10^{-5} M crystal violet and malachite green solutions.

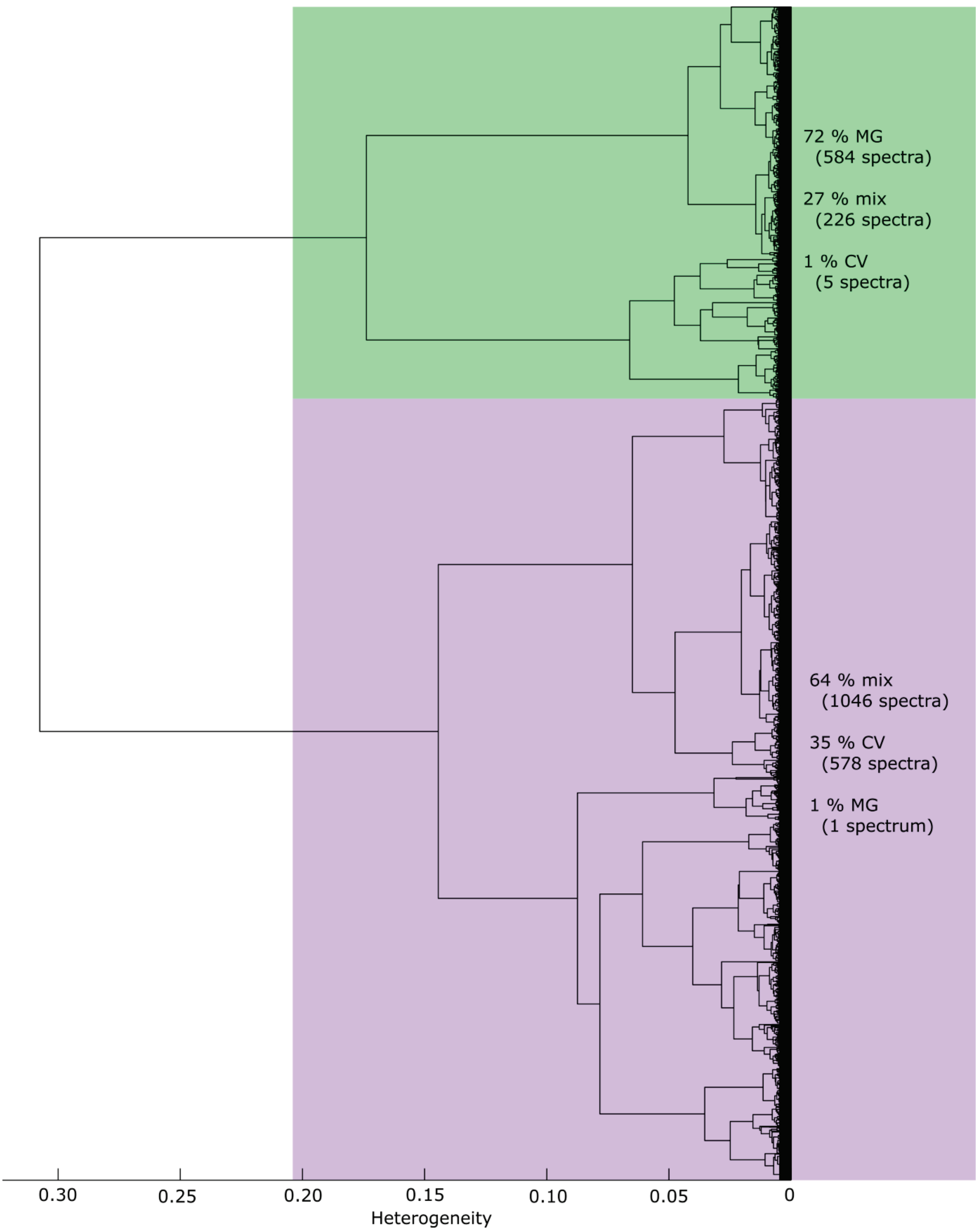

Fig. S2 Results of a hierarchical cluster analysis of the SEHRS spectra from the distinct regions of the MG/mix and CV/mix samples discussed in Figures 3 and 4 in the paper, of other samples prepared in the same way with different dye concentrations, and from solutions of the two dyes on immobilized silver nanoparticles. Cluster analysis was performed on vector-normalized first derivatives of the spectra in the range from 380-1000 cm^{-1} using Euclidean distance measure and Ward's algorithm for clustering. 'MG' refers to spectra from regions on the samples where only malachite green (MG) is present or to MG solutions, 'CV' refers to regions where only crystal violet (CV) is present, or to CV solutions, 'mix' refers to regions that were immersed in both MG and CV (see schematic in manuscript

Figure 1), or to solutions with equal concentrations of CV and MG. The percentages are based on the number of spectra forming each of the two respective clusters.
